# Controllable broadband multicolour single-mode polarized laser in a dye-assembled homoepitaxial MOF microcrystal

**DOI:** 10.1038/s41377-020-00376-7

**Published:** 2020-08-13

**Authors:** Huajun He, Yuanjing Cui, Hongjun Li, Kai Shao, Banglin Chen, Guodong Qian

**Affiliations:** 1 State Key Laboratory of Silicon Materials, Cyrus Tang Center for Sensor Materials and Applications, School of Materials Science and Engineering, Zhejiang University, 310027 Hangzhou, China; 2 Department of Chemistry, University of Texas at San Antonio, San Antonio, TX 78249 USA; 3 Present Address: Division of Physics and Applied Physics, School of Physical and Mathematical Sciences, Nanyang Technological University, 21 Nanyang Link, Singapore, 637371 Singapore

**Keywords:** Optical materials and structures, Lasers, LEDs and light sources

## Abstract

Multicolour single-mode polarized microlasers with visible to near-infrared output have very important applications in photonic integration and multimodal biochemical sensing/imaging but are very difficult to realize. Here, we demonstrate a single crystal with multiple segments based on the host-guest metal-organic framework ZJU-68 hierarchically hybridized with different dye molecules generating controllable single-mode green, red, and near-infrared lasing, with the lasing mode mechanism revealed by computational simulation. The segmented and oriented assembly of different dye molecules within the ZJU-68 microcrystal causes it to act as a shortened resonator, enabling us to achieve dynamically controllable multicolour single-mode lasing with a low three-colour-lasing threshold of ~1.72 mJ/cm^2^ (approximately seven times lower than that of state-of-the-art designed heterostructure alloys, as reported by Fan F et al. (Nat. Nanotechnol. 10:796–803, 2015) considering the single pulse energy density) and degree of polarization >99.9%. Furthermore, the resulting three-colour single-mode lasing possesses the largest wavelength coverage of ~186 nm (ranging from ~534 to ~720 nm) ever reported. These findings may open a new route to the exploitation of multicolour single-mode micro/nanolasers constructed by MOF engineering for photonic and biochemical applications.

## Dye-infused microcrystals for multicolour lasing

A three-colour laser built from microcrystals infused with several different dyes could be hugely beneficial in biochemical imaging. Metal-organic frameworks (MOFs), assembled from metal ions and organic ligands, contain engineered pores and channels in which guest molecules can be arranged in highly ordered structures for unique fluorescent emission. Guodong Qian and co-workers at Zhejiang University, China, and University of Texas, USA, infused three different linear dye molecules in an MOF called ZJU-68. When these crystals were illuminated with blue light, they emitted polarized light at three different wavelengths in green, red and near-infrared. This represents a wavelength band of 186 nanometres for three-colour single-mode lasing, the widest ever recorded. This type of highly-controllable multicolour lasing could not only improve biochemical sensing and imaging, but also provide routes towards rapid optical signal processing in miniaturized photonic devices.

## Introduction

Multicolour single-mode polarized laser sensing/imaging is a very promising diagnostic technology that has yet to be developed^1^ (Supplementary Fig. 1). First, a light source with broadband (visible to near-infrared) multicolour output provides the fundamental basis for multimodal/multidimensional sensing/imaging^2^ since different tissues, cells or biochemicals have different responses (optical^3–5^, thermal^6–8^, acoustic^9,10^, etc.) to different wavelengths of light. On the other hand, the polarization properties of light provide an opportunity for the analysis and processing of scattered light signals and can help obtain structural information rich in biological materials^11,12^. In addition, single-mode micro/nanolasers meet the application requirements of miniaturized photonic devices with high information accuracy, avoiding false signals and overlapping interference of different optical signals, with the potential to achieve target sensing/imaging of various cells and molecules when combined with multicolour output characteristics^13,14^. To utilize the uniqueness of broadband multicolour output, polarization and single-mode micro/nanolasing, monomeric multicolour polarized single-mode lasing micro/nanocrystals with controllable output from the visible to near-infrared (NIR) are potentially very useful for miniaturized multimodal sensing/imaging but have never been realized. To date, the use of semiconductor heterostructures with different gain bands has only achieved multicolour amplified spontaneous emission (ASE) or multimode lasing in the visible band without reports of polarization properties, which was often accompanied by severe spontaneous emission components^1,15–19^.

Among various active luminescent materials, organic luminescent materials can combine simple processing techniques with molecular diversity and material compatibility and are considered one of best gain media for manufacturing multiwavelength lasers ranging from the visible to NIR, but adverse interactions between organic molecules, such as aggregation-caused quenching (ACQ) and energy transfer phenomena caused by Förster resonance, make it difficult to achieve efficient and stable multicolour laser output^13,20^. The metal-organic framework (MOF), a periodic porous crystalline material assembled by metal ions and organic bridging ligands, is considered a powerful hybrid material platform and provides opportunities to solve the mentioned issues^21–25^. Its highly engineered pore/channel structure provides excellent spatial confinement to assemble and isolate guest organic molecules, which can reduce intermolecular interactions for effective inhibition of excited-state energy transfer and even control of output emission characteristics^26–28^. In addition, the smooth and regular crystal morphology of the MOF can enable it to effectively act as an optical resonator to provide optical feedback^29^. If guest organic molecules with different gain bands can be spatially separated by MOF crystal and pore/channel engineering while maintaining the efficient spatial confinement and orientation of the host MOF for the guest organic molecules, that is, ensuring an extremely high-gain concentration and oriented emission transition moment, then realization of a multicolour single-mode polarized laser with a low threshold in the shortened resonant cavity is expected.

Here, we demonstrate simultaneous in situ assembly of different guest dye molecules based on the homoepitaxy process of the host framework ZJU-68^26^ in an optimized host-guest hierarchical hybrid MOF single crystal system to achieve multicolour single-mode lasing. The MOF hierarchical hybrid single crystal exhibits different macroscopically segmented colours, which suggests that spatially segmented assembly of different dye molecules in a single MOF microcrystal has been achieved. The homoepitaxial growth of the host framework ZJU-68 ensures uniformity and high quality of the single monolithic cavity. The spatially segmented assembled, tightly confined and highly oriented cationic linear dye molecules (*E*)-1-methyl-4-(2-(1-methyl-1*H*-pyrrol-2-yl)vinyl)pyridinium (MMPVP), (*E*)-4-(4-(dimethylamino)styryl)-1-methylpyridinium (DMASM) and 4-((1*E*,3*E*)-4-(4-(dimethylamino)phenyl)buta-1,3-dienyl)-1-methylpyridinium (DPBDM) in the anionic ZJU-68 sub-nanochannels efficiently minimize the intermolecular energy transfer (in particular avoiding the severe energy transfer caused by overlap of the short-wavelength dye emission band and the long-wavelength dye absorption band), increase the gain concentration, and optimize the orientation of dye molecules within the framework, resulting in high-gain dynamically controllable multicolour single-mode lasing with a degree of polarization > 99.9% and a low three-colour-lasing threshold of ~1.72 mJ/cm^2^ in a shortened laser cavity. Combined with the lasing pattern of the sample, the computationally simulated electric field distribution reveals the possible lasing mode mechanisms of the three colours of light waves in the hybrid cavity and qualitatively explains their propagation differences. In addition, the largest multicolour single-mode laser emission wavelength coverage in a single monolithic structure has been observed, with a wavelength range of 186 nm (from visible light ~534 to NIR ~720 nm). The results demonstrate the unique strategy of spatially controlling the laser colour through regional assembly of a guest gain medium constructed via MOF crystal engineering and provide a new route for multicolour solid-state microlaser materials for photonic integration and multimodal biochemical sensing/imaging.

## Results

### Synthesis and characterization

**Fig. 1:**
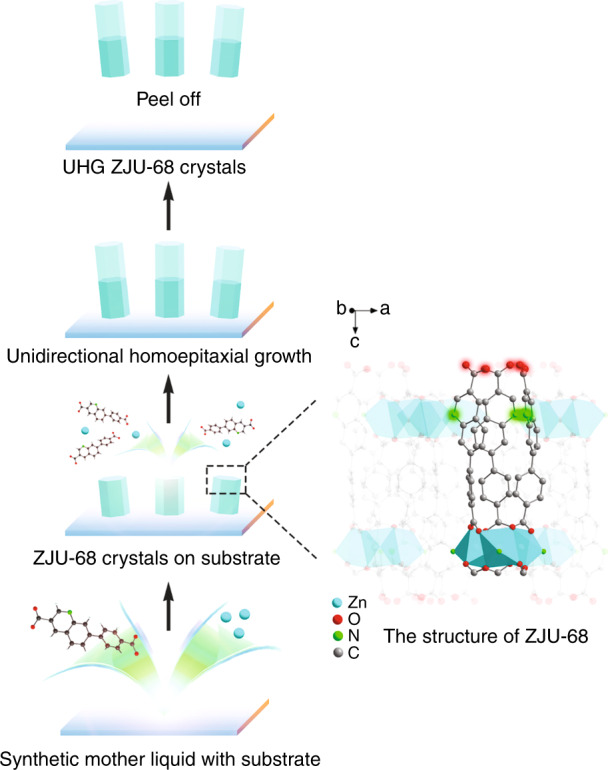
Schematic of uniaxial homoepitaxial growth of ZJU-68 crystals. The organic bridging ligand H_2_CPQC provides a higher chelating site density along the crystal axis direction, and the significant difference in the axial and radial chelation site densities makes the crystal tend to epitaxially grow along the axial direction in the growth solution with less H^+^. In addition, the introduction of the substrate prevents the one end face of the ZJU-68 crystal attached to the substrate from contacting the framework-building elements; thus, the epitaxial growth of the crystal has unidirectionality, and finally, uniaxially homoepitaxially grown (UHG) ZJU-68 crystals are obtained

**Fig. 2:**
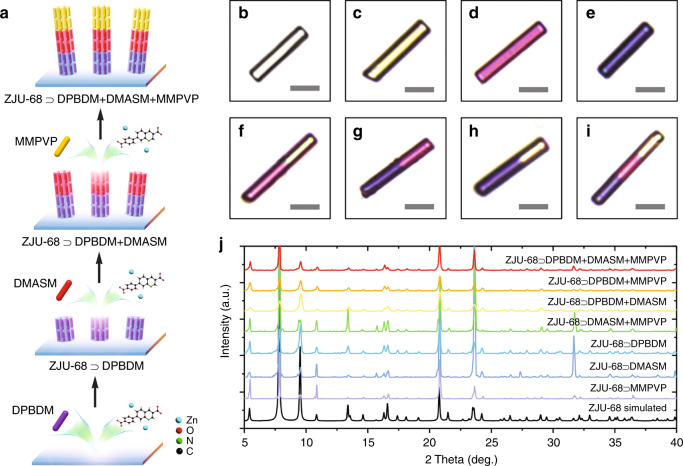
Synthesis and characterization of UHG hierarchical hybrid ZJU-68 microcrystals. **a** Schematic of the synthesis of UHG hierarchical dye-assembled hybrid ZJU-68 microcrystals. **b–i** Optical micrographs of ZJU-68 (**b**), ZJU-68⊃MMPVP (**c**), ZJU-68⊃DMASM (**d**), ZJU-68⊃DPBDM (**e**), ZJU-68⊃DMASM + MMPVP (**f**), ZJU-68⊃DPBDM + DMASM (**g**), ZJU-68⊃DPBDM + MMPVP (**h**), and ZJU-68⊃DPBDM + DMASM + MMPVP (i); scale bars, 10 μm. **j** PXRD patterns of ZJU-68 and hierarchical hybrid ZJU-68 microcrystals, which indicate that hierarchical hybrid ZJU-68 microcrystals have a framework structure identical to that of ZJU-68

Figure 1 schematically shows the newly developed method for fabricating uniaxially homoepitaxially grown (UHG) ZJU-68 metal-organic framework crystals. The ZJU-68 crystal is constructed by the organic ligand 7-(4-carboxyphenyl)quinoline-3-carboxylic acid (H_2_CPQC) and the trinuclear secondary building unit (SBU) [Zn_3_O]^4+^ formed by bridging Zn^2+^ and *μ*_3_-O^26^. Through the structural analysis of the host framework ZJU-68 (Fig. 1), it is known that the growth along the crystal axis is mainly ascribed to the coordination of the carboxyl groups at the two ends of the organic ligand with the SBU, while the growth in the radial direction mainly depends on the coordination of the nitrogen atom at the side end of the organic ligand with the SBU, whereby the axial coordination density is much higher than that in the radial direction from a spatial perspective. When the pH of the reaction solution is lowered, H^+^ preferentially coordinates with the carboxyl groups at both ends of the ligand, thereby delaying the growth of the ZJU-68 crystal along the crystal axis direction, and the crystal tends to form massive particles. Conversely, elimination of the use of an additional acid is beneficial to the growth of the ZJU-68 crystal along the crystal axis, which not only contributes to shortening of the cavity length, facilitating the acquisition of single-mode lasing, but also effectively controls the ZJU-68 crystal tendency to homoepitaxially grow along the crystal axis direction. Furthermore, at the beginning of the preparation, the introduction of the substrate led to one end face of a considerable portion of the ZJU-68 crystals grown in the first step adhering to the substrate, preventing it from contacting the framework-constructing elements in the subsequent homoepitaxial growth, thereby making the homoepitaxial growth of the crystal unidirectional instead of bidirectional, and finally, uniaxially homoepitaxially grown ZJU-68 crystals were obtained. Combined with the advantages of spatial confinement of MOF pores/channels, this approach of controlling the unidirectional homoepitaxial growth of MOF crystals brings about opportunities for spatially ordered assembly of different guest materials. As such, the dye-assembled hierarchical hybrid metal-organic framework structure was synthesized by the combination of in situ assembly and homoepitaxial growth mentioned above. The synthesis procedure is shown schematically in Fig. 2a. First, in situ self-assembly of Zn^2+^, organic linker H_2_CPQC and one kind of dye molecule (denoted dye-1) in *N*,*N*-dimethylformamide/acetonitrile at 100 °C afforded monochromatic hexagonal prism microcrystals^26^, denoted ZJU-68⊃dye-1. Second, the resulting microcrystals were then immersed into a new reaction solution of Zn^2+^ and H_2_CPQC with another kind of dye molecule (denoted dye-2) to self-assemble two-colour hierarchical hybrid microcrystals, denoted ZJU-68⊃dye-1+dye-2. Third, repeating the second step, three-colour or multicolour hierarchical hybrid microcrystals ZJU-68⊃dye-1+dye-2 + ···+dye-*n* could be obtained (dye-*n* denotes the dye molecule used in the *n*^th^ step). Three different linear dye molecules were used in this study, namely, (*E*)-1-methyl-4-(2-(1-methyl-1*H*-pyrrol-2-yl)vinyl)pyridinium (MMPVP), (*E*)-4-(4-(dimethylamino)styryl)-1-methylpyridinium (DMASM) and 4-((1*E*,3*E*)-4-(4-(dimethylamino)phenyl)buta-1,3-dienyl)-1-methylpyridinium (DPBDM). Through the combination of these three different dye molecules, seven different hybrid MOF single crystals could be obtained (Fig. 2c–i). These hybrid single crystals all have a hexagonal prismatic crystal morphology similar to that of pure ZJU-68 (Fig. 2b), except for the colour change due to the assembly of the dye molecules. The colours corresponding to the crystal segments in which the dyes MMPVP, DMASM, and DPBDM are assembled are light yellow, magenta, and purple, respectively. The as-synthesized dye-assembled ZJU-68 microcrystals show magenta-yellow/purple-magenta/purple-yellow two-segment (Fig. 2f-h) and purple-magenta-yellow three-segment hierarchical structures (Fig. 2i) under bright field, confirming the successful in situ assembly and homoepitaxial growth of the MOFs.

Figure 2j shows the powder X-ray diffraction (PXRD) patterns of dye-assembled ZJU-68 hierarchical hybrid microcrystals. The results show that the PXRD patterns of these hybrid crystals have the same peak positions as the simulated PXRD spectrum of pure ZJU-68^26^, and there is no redundant peak, indicating that the structures of the as-synthesized hybrid crystals are pure and that the host ZJU-68 framework still maintains the initial crystal structure after assembly of different dye molecules and homoepitaxial growth. Since a fresh and concentration-determined solution is used for the homoepitaxial growth of each new crystal segment, the assembly concentrations of the dyes in the ZJU-68 crystal can be further determined by separately synthesizing monochromatic hybrid crystals of the specific dye molecules. Accordingly, as calculated by ^1^H NMR analysis (NMR = nuclear magnetic resonance), per gram, the resulting ZJU-68⊃MMPVP crystals contain 80.8 mg of dye molecules, corresponding to a concentration of 0.67 M (the molar amount of dye per unit volume of the solid crystal) (Supplementary Fig. 2), which results in the corresponding chemical formula of the ZJU-68⊃MMPVP segment being (MMPVP)_0.48_H_1.52_[Zn_3_O(CPQC)_3_]. The dye assembly concentrations of ZJU-68⊃DMASM and ZJU-68⊃DPBDM can be obtained by the same method (Supplementary Fig. 3 and Supplementary Fig. 4), which are ~0.44 M and ~0.53 M, and the corresponding chemical formulas are (DMASM)_0.32_H_1.68_[Zn_3_O(CPQC)_3_] and (DPBDM)_0.38_H_1.62_[Zn_3_O(CPQC)_3_], respectively. Homoepitaxy does not require consideration of matching of the lattice parameters and is conducive to ensuring the consistency of the crystal growth direction before and after the epitaxy process, which means that the direction of the one-dimensional sub-nanochannels of ZJU-68 is always along the crystal axis. The width of the linear guest dye molecules used in the study is matched to the channel diameter of ZJU-68, and they all have a single absorption/emission transition moment, which is oriented along the length of the molecule. Therefore, through the homoepitaxial in situ self-assembly strategy using the host framework ZJU-68, different linear dye molecules can be hierarchically assembled in ZJU-68 along the same direction, forming an oriented and hierarchical guest molecular integration system.

### Multicolour fluorescence

**Fig. 3:**
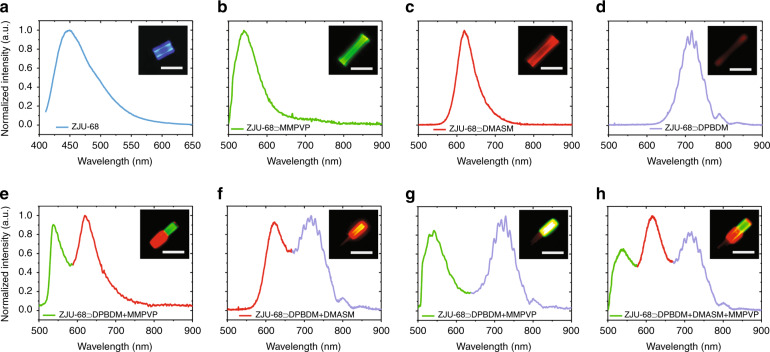
Fluorescence of UHG hierarchical hybrid ZJU-68 microcrystals. **a** Fluorescence spectrum of a single ZJU-68 microcrystal excited at 390 nm. **b**–**h** Fluorescence spectra of single hierarchical hybrid microcrystals ZJU-68⊃MMPVP (**b**), ZJU-68⊃DMASM (**c**), ZJU-68⊃DPBDM (**d**), ZJU-68⊃DMASM + MMPVP (**e**), ZJU-68⊃DPBDM + DMASM (**f**), ZJU-68⊃DPBDM + MMPVP (**g**), and ZJU-68⊃DPBDM + DMASM + MMPVP (**h**) excited at 480 nm. Insets: fluorescence micrographs of different hierarchical hybrid ZJU-68 microcrystals. Scale bars, 10 μm

Figure 3 compares the photoluminescence (PL) spectra of dye-assembled ZJU-68 hybrid crystals. It can be seen from the characteristics of the PL spectra that after assembly of the dye molecules, the fluorescence emission from the crystals is entirely converted from the blue-light emission of pure ZJU-68 (peak at ~450 nm, see Fig. 3a) to the characteristic emission colours of the dyes. Under excitation by a mercury lamp with a 480 nm excitation filter set, ZJU-68⊃MMPVP, ZJU-68⊃DMASM and ZJU-68⊃DPBDM show green, red, and near-infrared emission with peaks at ~540, ~620, and ~716 nm, respectively (Fig. 3b–d). The corresponding full widths at half maximum (FWHMs) are 70.5, 54.7, and 61.0 nm. The reasons for choosing 480 nm as the excitation wavelength are as follows: (1) The optimal absorption position of the dye DMASM is near 470 nm^30^, and the remaining two dye molecules can be effectively excited by 480 nm^31^. (2) The 480 nm wavelength light is located in the emission band of ZJU-68 and is basically not absorbed by ZJU-68 (see Supplementary Fig. 5), which is conducive to promoting the population inversion process of the dye molecules by avoiding the optical loss caused by the host-guest energy transfer process. (3) Compared with ultraviolet light, 480 nm light causes less damage to organic molecules, which is conducive to the stability of the resulting luminescence performance. For the homoepitaxial hybrid MOF structures in which different dye molecules are assembled in different crystal segments, the emission of different colours is spatially segmented under excitation at 480 nm (Fig. 3e–h). The emission peak positions of these different colour segments are substantially coincident with the emission peak positions of the MOF structures assembled by the monochromatic dyes. Taking ZJU-68⊃DPBDM + DMASM + MMPVP as an example (Fig. 3h), the fluorescence emission spectrum has three peak positions, namely, 540, 619, and 716 nm, corresponding to the three-segment assembly of MMPVP, DMASM, and DPBDM, and there is no significant peak shift phenomenon. It is worth noting that for the hybrid crystals shown in Fig. 3, when the emission detection direction is perpendicular to the crystal axis, almost no fluorescence signal can be observed upon excitation. This is because the MMPVP, DMASM, and DPBDM linear molecules can be orderly aligned in the interior of the framework by utilizing the size-matched one-dimensional channels of ZJU-68^26,28^. The alignment of the emission transitions of these dye molecules thus results in significant emission anisotropy. Multichannel confocal laser scanning microscopy also clearly shows that the hybrid single crystal ZJU-68⊃DPBDM + DMASM + MMPVP can be combined with different incident light and filter modules to obtain segmented excitation and different colour fluorescence signal output (Supplementary Fig. 6). The definite segmented fluorescence boundaries indicate that the ZJU-68 homoepitaxial crystal is substantially grown along the crystal axis direction, which is different from the core-shell epitaxial growth of most MOF crystals^32,33^. This should be mainly due to the number and density of coordination sites in the axial direction of the ZJU-68 structure being much larger than those in the radial direction. Through the combination of self-assembly and homoepitaxial methods, the advantages of efficient spatial confinement and spatial segmentation of different dye molecules provided by ZJU-68 are successfully integrated, effectively avoiding the aggregation-caused quenching (ACQ) effect and the energy transfer from short-wavelength dye molecules to long-wavelength dye molecules, which is conducive to efficient multiwavelength emission output.

### Multicolour lasing performance

**Fig. 4:**
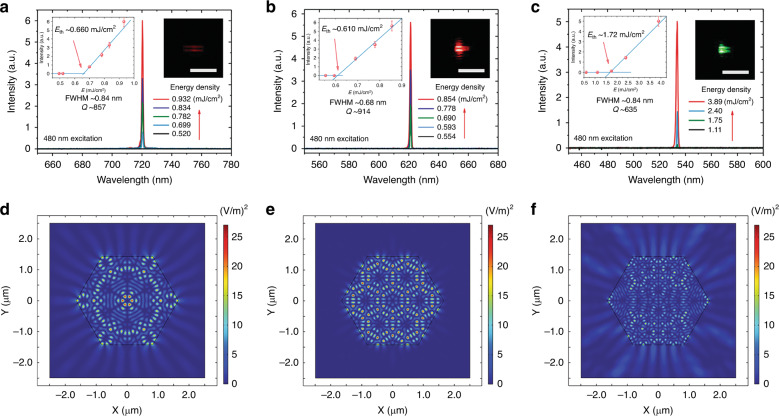
Single-mode lasing in an individual ZJU-68⊃DPBDM + DMASM + MMPVP hierarchical hybrid microcrystal (*R* ~1.65 μm). **a**–**c** Emission spectra of ZJU-68⊃DPBDM (**a**), ZJU-68⊃DMASM (**b**), and ZJU-68⊃MMPVP (**c**) crystal segments around the lasing threshold. Insets: micrographs of ZJU-68⊃DPBDM (**a**), ZJU-68⊃DMASM (**b**) and ZJU-68⊃MMPVP (**c**) crystal segments excited at 480 nm (right), and emission intensity as a function of pump fluence showing a lasing threshold of ~0.660 mJ/cm^2^ for ZJU-68⊃DPBDM, ~0.610 mJ/cm^2^ for ZJU-68⊃DMASM, and ~1.72 mJ/cm^2^ for ZJU-68⊃MMPVP (left). Scale bars, 10 μm. **d**–**f** Simulated electric field distributions (square of the electric field intensity) in the hexagonal cavity for the 720 nm (**d**), 621 nm (**e**), and 534 nm (**f**) modes

The lasing property was investigated for an individual small hybrid crystal of ZJU-68⊃DPBDM + DMASM + MMPVP with a side length (*R*) of ~1.65 μm under a microscope at room temperature. A 480-nm laser beam from a femtosecond optical parametric amplifier (OPA) was coupled into the microscope. The PL signal of the test sample was collected by a fibre optic spectrometer. Figure 4 shows the representative PL spectra of the isolated hybrid crystal near the lasing threshold. The hierarchical hybrid single crystal ZJU-68⊃DPBDM + DMASM + MMPVP is divided into three sections, which correspond to the ZJU-68⊃DPBDM crystal segment, ZJU-68⊃DMASM segment, and ZJU-68⊃MMPVP segment. For the crystal segment in which the DPBDM molecule is assembled (Fig. 4a), the fibre optic spectrometer shows no substantial luminescence signal under excitation at a low pump fluence (*E*) of ≤0.520 mJ/cm^2^. The pump fluence is defined as the laser energy density directly received by the MOF crystal (after going through the objective lens and before being incident on the MOF crystal). As the pump energy density increases to ≥0.699 mJ/cm^2^, a narrow peak centred at ~720 nm appears and increases rapidly as the pump fluence increases, while the intensity of the broad spontaneous emission remains almost constant. The crystal photomicrograph taken during the pumping process (the right inset of Fig. 4a) shows bright dark-red striped patterns on both sides of the hexagonal prism crystal, which can be ascribed to the whispering gallery mode (WGM) mechanism as discussed in our previous work^28^. The left inset in Fig. 4a illustrates the pump fluence dependence of the emission intensity, indicating a linear relationship with the pump fluence and a low lasing threshold of *E*_th_ ~0.660 mJ/cm^2^. The full width at half maximum (FWHM) of the pinpoint narrow peak is ~0.84 nm, which is two orders of magnitude narrower than that (61.0 nm) of the fluorescent emission band of ZJU-68⊃DPBDM. The presence of a single peak accompanied by a significant spectral narrowing and the threshold energy coupled with the linear rapid increase in intensity with the pump fluence suggest that single-mode lasing has occurred in the ZJU-68⊃DPBDM crystal segment. The quality factor (*Q*) is given by *Q* = *λ*/δ*λ*, where *λ* and δ*λ* are the peak wavelength and FWHM, respectively. At a pump fluence of 0.834 mJ/cm^2^, the FWHM of the lasing peak at 720 nm is ~0.84 nm, and the crystal segment can be deduced to have a quality factor of *Q* ~857 for the laser cavity. The crystal segment ZJU-68⊃DMASM (Fig. 4b) or ZJU-68⊃MMPVP (Fig. 4c) assembled with DMASM or MMPVP molecules can also achieve single-mode lasing operation, showing a bright red or bright green stripe-spot pattern with the spike-like spectrum peaking at 621 nm or 534 nm during pumping. The emission intensity as a function of pump fluence shows that their lasing thresholds are ~0.610 mJ/cm^2^ and ~1.72 mJ/cm^2^, and the quality factors *Q* are calculated as ~914 and ~635, respectively. Compared to the crystal segments in which DMASM or DPBDM are assembled, the higher threshold and lower *Q* of the ZJU-68⊃MMPVP crystal segment may be attributed to the weaker lasing efficiency of MMPVP resulting in a higher pump energy density, and the high-energy photons at a high pump fluence will affect the quality of the cavity. Although the results show that the three-colour lasing can be attributed to the WGM mechanism, basically the same cavity will have different effects on the propagation and feedback of different wavelengths within it. To reveal these possible differences and the more detailed lasing mode mechanisms in the hierarchical hybrid hexagonal cavity, qualitative optical simulations were further conducted in an eigenfrequency study by using COMSOL Multiphysics (version 5.4). Figure 4d–f shows the computed electric field distributions (square of the electric field intensity) in the hierarchical hybrid hexagonal cavity for analysed mode wavelengths of 720, 621, and 534 nm. Compared with the distribution of red light (621 nm) and green light (534 nm), the distribution of near-infrared light (720 nm) in the cavity is more concentrated at the corners of the hexagonal cavity, which matches the lasing pattern in optical images (insets in Fig. 4a–c) very well. For the crystal (cavity) size used in the experiment (*R* = 1.65 μm), red light with a wavelength of 621 nm shows a more concentrated and stronger optical field distribution, while the simulation result corresponding to 534 nm green light is the most disperse and weak. In addition, the characteristics of the WGM mechanism, that is, the internal reflection of the six crystal facets in turn, can be clearly seen in the simulated diagrams for the NIR lasing at 720 nm (Fig. 4d) and the red lasing at 621 nm (Fig. 4e). However, the simulation diagram for the green lasing at 534 nm (Fig. 4f) shows few such characteristics, indicating that the WGM mechanism has not been steadily established in this case. These results suggest that the cavity is more conducive to the formation of a stable standing wave mode for red light at 621 nm and NIR light at 720 nm such that the light loss is lower and the *Q* value is greater, contributing to the lower thresholds, while the result for green light is the opposite, which corresponds to the experimental data. Considering the single pulse energy density, the three-colour-lasing threshold of the resulting ZJU-68⊃DPBDM + DMASM + MMPVP hybrid single crystal is ~1.72 mJ/cm^2^, which is ~7 times lower than that of the state-of-the-art designed heterostructure alloy ZnCdSSe for three-colour lasing reported previously^1^. A comparison of the lasing thresholds of multicolour lasing monomer materials reported thus far^1,15–17,19,27,31,34^ can be found in Supplementary Table 1. Although the threshold has a relatively high peak power density compared with some reported multicolour lasing monomer materials due to the narrow excitation pulse width, it is foreseeable that the lasing threshold of the resulting ZJU-68⊃DPBDM + DMASM + MMPVP can be further reduced by means such as thin metal layer coating^35^. Combining the advantages of a wide and controllable emission range, a single mode, a narrow line width and high linear polarization output (see details below), the resulting ZJU-68⊃DPBDM + DMASM + MMPVP still has high competitiveness compared with existing monolithic multicolour lasing materials (see Supplementary Table 2). In addition, most of the reported multicolour lasing was still accompanied by obvious or severe spontaneous emission components^1,16,17^, which indicates the difficulty of constructing an excellent cavity for multicolour lasing to a certain degree. The high-quality factor, acceptably low multicolour lasing threshold and highly polarized emission with almost pure stimulated emission of ZJU-68⊃DPBDM + DMASM + MMPVP should be attributed to the efficient spatial confinement effect provided by host metal-organic framework ZJU-68 and the fact that the smooth and regular crystal morphology of ZJU-68 is maintained while assembling different dye molecules during the epitaxial growth process.

**Fig. 5:**
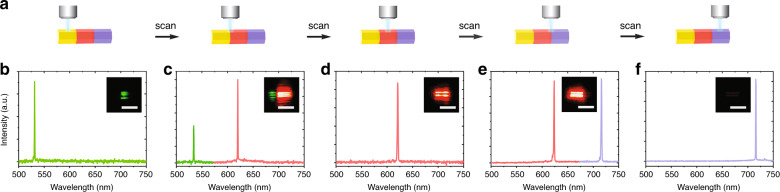
Scanning lasing performance in a single ZJU-68⊃DPBDM + DMASM + MMPVP hierarchical hybrid microcrystal. **a** Schematic illustration of a hierarchical hybrid structure excited with a scanning focused laser beam. **b**–**f** Corresponding lasing spectra collected from the five different excitation areas shown in (**a**). Scale bars, 10 μm

The MOF homoepitaxial structure obtained by hierarchically assembling different dye molecules provides the opportunity for scanning multicolour lasing. Taking the ZJU-68⊃DPBDM + DMASM + MMPVP single microcrystal as an example, Fig. 5a illustrates the scanning pump lasing in different regions of the microcrystal along the crystal axis direction. Each part of the hierarchical structure is selectively focused on and excited by a femtosecond laser (480 nm) that passes through an objective lens, and the corresponding lasing spectra are shown in Fig. 5b–f. The excitation position is initially at one end of the hybrid crystal, passes through the junction of two crystal segments, then proceeds to the next segment, and so on. Each crystal segment exhibits a sharp single peak at approximately 531 (Fig. 5b), 620 (Fig. 5d), and 716 nm (Fig. 5f) upon excitation, corresponding to green, red, and near-infrared (NIR) emission. When the pump position is at the junction of crystal segments, the two crystal segments are simultaneously excited to obtain bright green/red (Fig. 5c) or red/NIR lasing (Fig. 5e), corresponding to the emission spectrum of the two single peaks, and the peak positions have no obvious displacement. The unique structure obtained by hierarchically assembling different dye molecules allows a laser of a specific colour or a combination of colours to be controllably outputted in a micro-nanospace, potentially for use in many fields, such as flow biochemical sensing and on-chip optical signal transmission and processing.

**Fig. 6:**
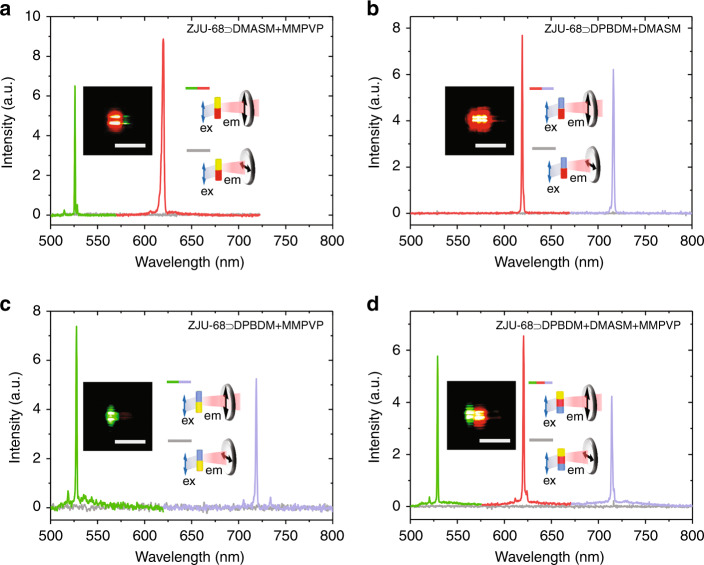
Tuneable multicolour polarized lasing performance in hierarchical hybrid ZJU-68 microcrystals. **a**–**d** Intensity-dependent emission spectra from individual hierarchical hybrid microcrystals ZJU-68⊃DMASM + MMPVP (**a**), ZJU-68⊃DPBDM + DMASM (**b**), ZJU-68⊃DPBDM + MMPVP (**c**), and ZJU-68⊃DPBDM + DMASM + MMPVP (**d**) with the emission detected at two polarization angles of *θ* = 0° (parallel to the crystal channels) and *θ* = 90° (perpendicular to the crystal channels), excited at 480 nm. Insets: Schematic diagrams of the measurement geometry for an individual crystal, and micrographs of hierarchical hybrid ZJU-68 microcrystals excited at 480 nm. Scale bars, 10 μm

Figure 6 shows the anisotropic lasing performance of dye-assembled hierarchical hybrid crystals, where the polarization direction of the pump light (480 nm, 2.01 mJ/cm^2^) is fixed direction parallel to the crystal axis. The PL signal from the sample first passes through a polarizer and then is collected by a fibre optic spectrometer. When the emission detection polarization direction is parallel to the crystal axis, the two-colour hierarchical hybrid crystals ZJU-68⊃DMASM + MMPVP (Fig. 6a), ZJU-68⊃DPBDM + DMASM (Fig. 6b), and ZJU-68⊃DPBDM + MMPVP (Fig. 6c) display dual-wavelength single-mode lasing spectra, accompanied by intense red/green, red/dark-red, and green/dark-red stripe-spot patterns, respectively. Among them, the single-mode lasing peak corresponding to the green light is located near 530 nm, the red light peak is at ~620 nm, and the dark-red light (near-infrared) peak is at ~716 nm. When the emission detection polarization direction is perpendicular to the crystal axis, a light emission signal can hardly be observed (consistent with the case of the monochromatic hybrid crystal ZJU-68⊃MMPVP, ZJU-68⊃DMASM, or ZJU-68⊃DPBDM, see Supplementary Fig. 7), which indicates that the emission has strong polarization, and the polarization direction is substantially oriented along the crystal axis. It is worth emphasizing that three-wavelength single-mode lasing is successfully realized in the three-colour hierarchical hybrid crystal ZJU-68⊃DPBDM + DMASM + MMPVP, and the single-mode lasing peak positions have no obvious displacement compared with the above data. Significant lasing light polarization is also observed in this system (Fig. 6d). The degree of polarization can be defined as DOP = (*I*_∥_ − *I*_⊥_)/(*I*_∥_ + *I*_⊥_) in our experiments (*I*_∥_ or *I*_⊥_ denotes the emission intensity detected when the emission detection polarization direction is parallel or perpendicular to the crystal axis, respectively)^35^, and the DOP of the above multicolour single-mode lasing can be calculated to be >99.9% (limited by the spectrometer sensitivity of our measurement system), demonstrating the first example of an inherently polarized multicolour lasing microcrystalline material. In addition, when the polarization direction of the pump light is perpendicular to the crystal axis, the dye-loaded ZJU-68 microcrystal can hardly be excited effectively; therefore, nearly no emission signals can be observed. The sensitive response to the excitation light polarization as well as inherently polarized lasing is attributed to the highly oriented assembly of the linear dye molecules within the one-dimensional channels of the host framework ZJU-68 microcrystal given that the absorption/emission transition moments of the linear dye molecules MMPVP, DMASM, and DPBDM are basically oriented along the molecular axis. These anisotropic multicolour lasing results indicate great potential for biochemical sensing/imaging, as well as optical signal processing in optoelectronic/photonic integration.

## Discussion

In summary, we have reported hierarchical assembly of different dye molecules based on a homoepitaxy process in a host-guest hybrid MOF microresonator to achieve up to three-wavelength single-mode lasing. An isomorphic cavity system with optimized segmented multicolour gain in axial space is constructed by simultaneously assembling different dye molecules in situ with the homoepitaxial host framework ZJU-68. The segmented assembly of different dye molecules not only spatially manipulates the colour output of the microlaser but also effectively solves the adverse effects of energy transfer between different dye molecules, especially for the systems with strong energy transfer in which the emission band of a short-wavelength dye substantially overlaps with the absorption band of a long-wavelength dye, thereby broadening the output band of the dye-based multicolour microlaser. In addition, owing to the high-efficiency spatial confinement effect and the regular smooth crystal morphology provided by ZJU-68, enabling the system to act as a high-quality resonator, the resulting three-colour lasing exhibits perfectly polarized emission, a low lasing threshold (single pulse energy density), a high-quality factor and almost pure stimulated emission compared to existing single monolithic materials for multicolour lasing. Furthermore, three-colour single-mode lasing over a wavelength range of 186 nm (from visible ~534 nm to NIR ~720 nm) in a single monolithic structure, the largest ever reported, has been observed. Our work greatly simplifies the process of creating a monolithic single-mode laser structure with dynamically controllable emission from the visible to NIR. It is foreseeable that these results will provide a reference for multimodal biochemical sensing/imaging and on-chip photonic information processing.

## Materials and methods

### Synthesis of ZJU-68⊃DPBDM + DMASM + MMPVP

A mixture of DPBDM iodide (5.44 μmol, ~2.1 mg), Zn(BF_4_)_2_·xH_2_O (0.068 mmol, ~20 mg), H_2_CPQC (0.034 mmol, 10 mg), DMF (2 mL), MeCN (0.4 mL), and H_2_O (0.01 mL) was sealed in a 15 mL Teflon-lined stainless-steel bomb, subjected to ultrasonic vibration for 5 min, and then heated at 100 °C for 24 h. After cooling to room temperature, the reacted solution was carefully removed, the purple crystals were washed three times with fresh DMF, and the DMF wash was thoroughly removed. A mixture of DMASM iodide (5.44 μmol, ~2.0 mg), Zn(BF_4_)_2_·xH_2_O (0.068 mmol, ~20 mg), H_2_CPQC (0.034 mmol, 10 mg), DMF (2 mL), MeCN (0.4 mL), and H_2_O (0.01 mL) was sealed in the above Teflon-lined stainless-steel bomb containing the washed purple crystal product, which was then heated at 100 °C for 6 h. After cooling to room temperature, the reacted solution was carefully removed, the purple-red crystals were washed three times with fresh DMF, and the DMF wash was thoroughly removed. A mixture of MMPVP iodide (5.44 μmol, ~1.8 mg), Zn(BF_4_)_2_·xH_2_O (0.068 mmol, ~20 mg), H_2_CPQC (0.034 mmol, 10 mg), DMF (2 mL), MeCN (0.4 mL), and H_2_O (0.01 mL) was sealed in the above Teflon-lined stainless-steel bomb containing the washed purple-red crystal product, which was then heated at 100 °C for 6 h. After cooling to room temperature and decanting the mother liquor, the purple-red-yellow segmented hexagonal crystalline product was rinsed four times with fresh DMF (5 mL × 4) and dried in air.

### Measurements

For lasing experiments, an optical parametric amplifier (Spirit-OPA + Spirit-OPA-UV3, Newport Corporation) was pumped by a fully automated ultrafast laser system (Spirit One 1040–8, 8 W at 1040 nm, Newport Corporation), which was used for generating the excitation pulse (1 kHz, 480 nm, pulse width < 400 fs). The incident laser was coupled to a microscope (IX71, Olympus) for focusing on crystals through an objective lens (Olympus LUCPlanFL N ×40, numerical aperture = 0.60). The PL signal from the sample was then focused and collected by a fibre optic spectrometer (PG2000-Pro, Ideaoptics Instruments).

## Supplementary information

Supplemental Information for Controllable broadband multicolour single-mode polarized laser in a dye-assembled homoepitaxial MOF microcrystal
