# Supplementary material for: Controllable broadband multicolour single-mode polarized laser in a dye-assembled homoepitaxial MOF microcrystal
Source: Light Sci Appl. 2020 Aug 13;9:138. doi: 10.1038/s41377-020-00376-7 (PMC7424519; doi:10.1038/s41377-020-00376-7)
Supplement: Supplementary file 1 — Supplemental Information for Controllable broadband multicolour single-mode polarized laser in a dye-assembled homoepitaxial MOF microcrystal [file 41377_2020_376_MOESM1_ESM.docx]

**Supplementary Information for**

**Controllable broadband multicolour single-mode polarized laser in a dye-assembled homoepitaxial MOF microcrystal**

Huajun He^1^ǂ†, Yuanjing Cui^1^†, Hongjun Li^1^, Kai Shao^1^, Banglin Chen^2^ and Guodong Qian^1^*

^1^ *State Key Laboratory of Silicon Materials, Cyrus Tang Center for Sensor Materials and Applications, School of Materials Science and Engineering, Zhejiang University, Hangzhou 310027, China*

^2^ *Department of Chemistry, University of Texas at San Antonio, San Antonio, TX 78249, USA*

ǂ *Present address: Division of Physics and Applied Physics, School of Physical and Mathematical Sciences, Nanyang Technological University, 21 Nanyang Link, Singapore 637371, Singapore*

† *These authors contributed equally to this article*

^*^Correspondence authors:

Guodong Qian

Email: [gdqian@zju.edu.cn](mailto:gdqian@zju.edu.cn)


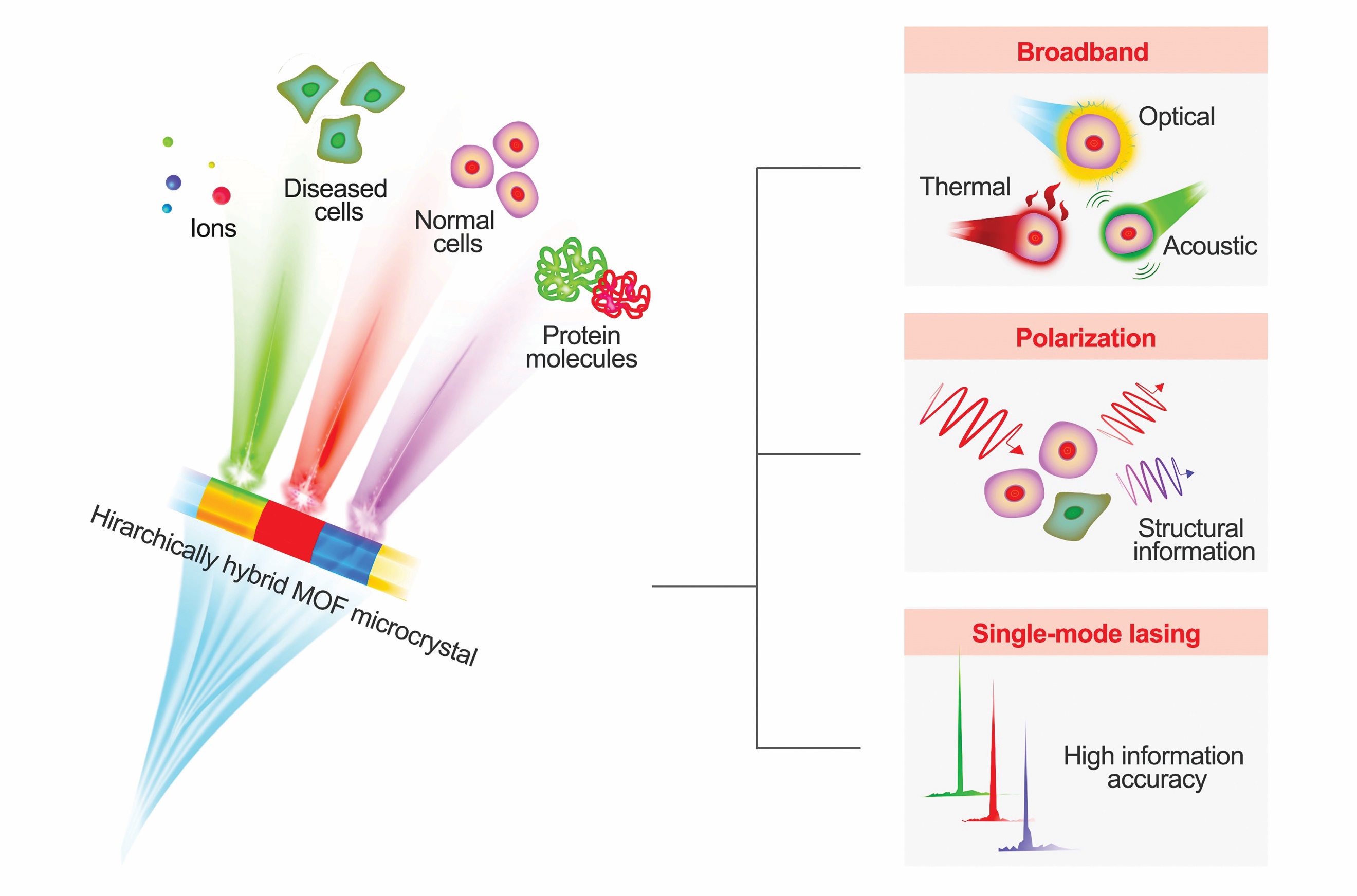


**Figure S1.** The broadband multicolor polarized single-mode lasing can potentially be used in multimodal biochemical sensing and imaging. Such unique lasing performance integrates the advantages of broadband output, polarization, and single-mode lasing, achieved by dye-assembled homoepitaxial hierarchically hybrid MOF microcrystal.


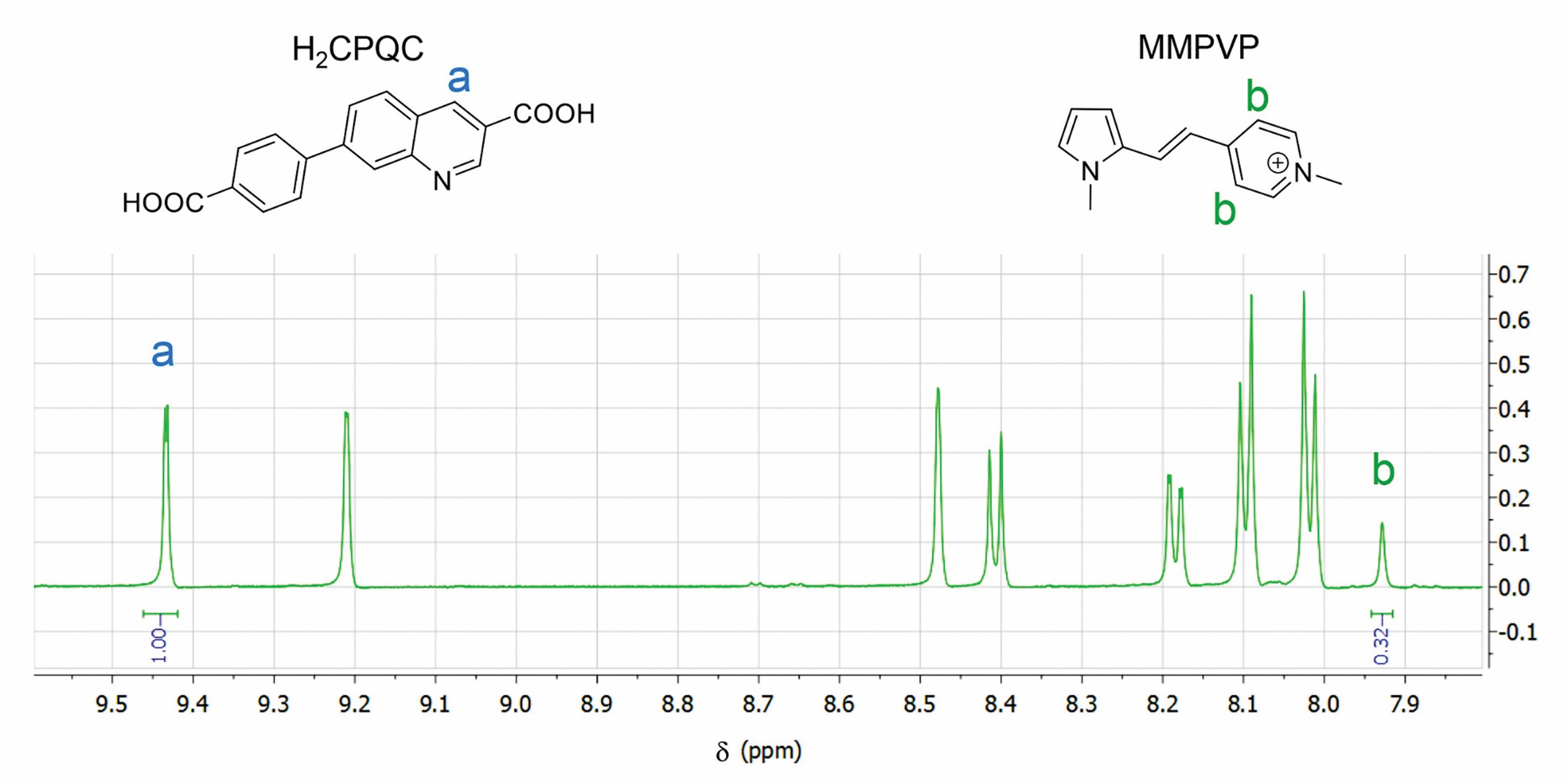


**Figure S2.** The partial ^1^H NMR spectrum of ZJU-68⊃MMPVP crystal segments. We calibrated and obtained peak area values of peaks that belong to linker H_2_CPQC and dye molecule MMPVP, respectively. The ratio (*R*_a_) of their peak area values represents the ratio of their concentrations in the crystal. The dye concentration of the ZJU-68⊃MMPVP crystal segment is calculated to be ~8.08 wt% (~0.67 M).


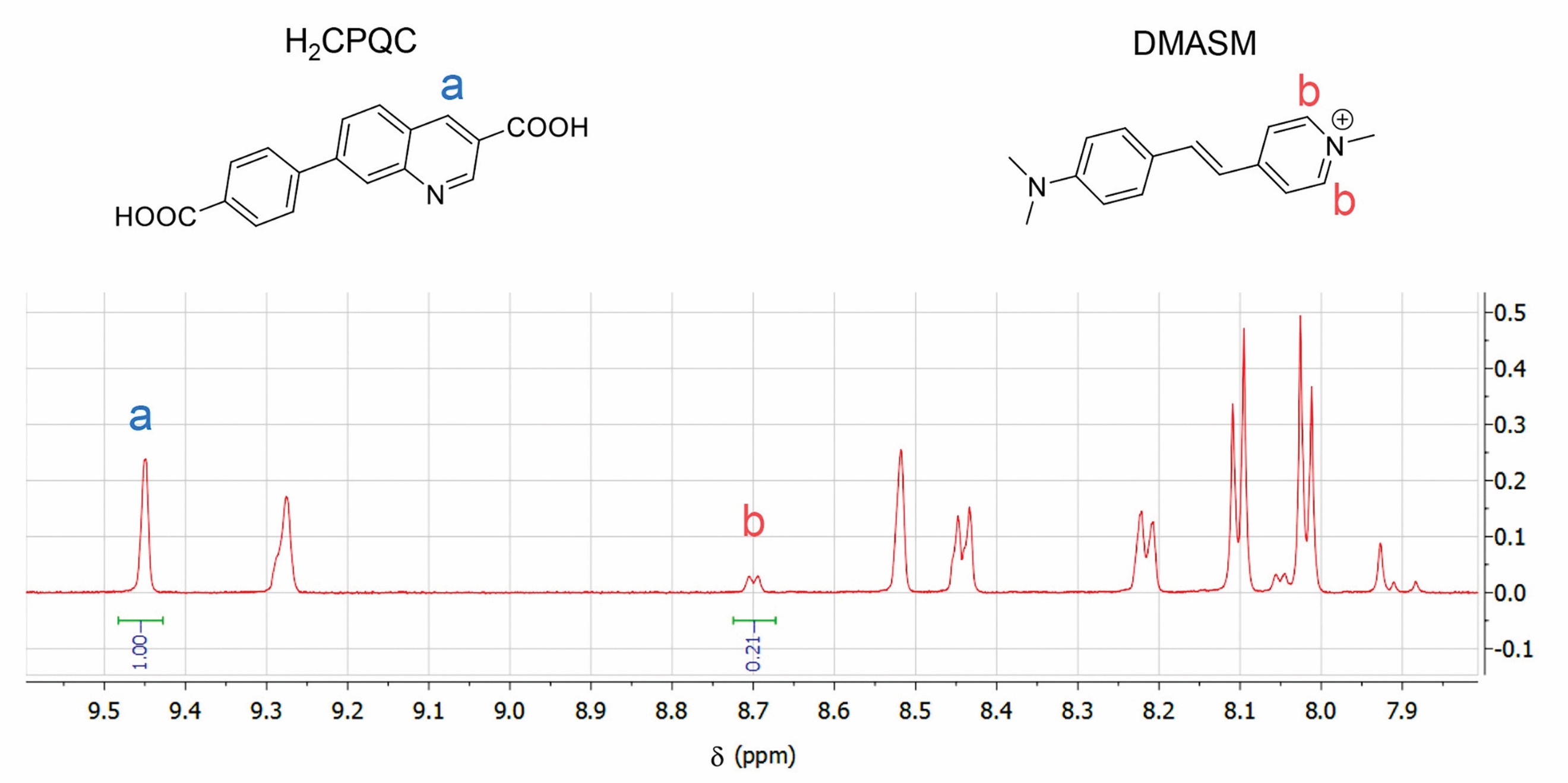


**Figure S3.** The partial ^1^H NMR spectrum of ZJU-68⊃DMASM crystal segments. We calibrated and obtained peak area values of peaks that belong to linker H_2_CPQC and dye molecule DMASM, respectively. The ratio (*R*_a_) of their peak area values represents the ratio of their concentrations in the crystal. The dye concentration of the ZJU-68⊃DMASM crystal segment is calculated to be ~6.58 wt% (~0.44 M).


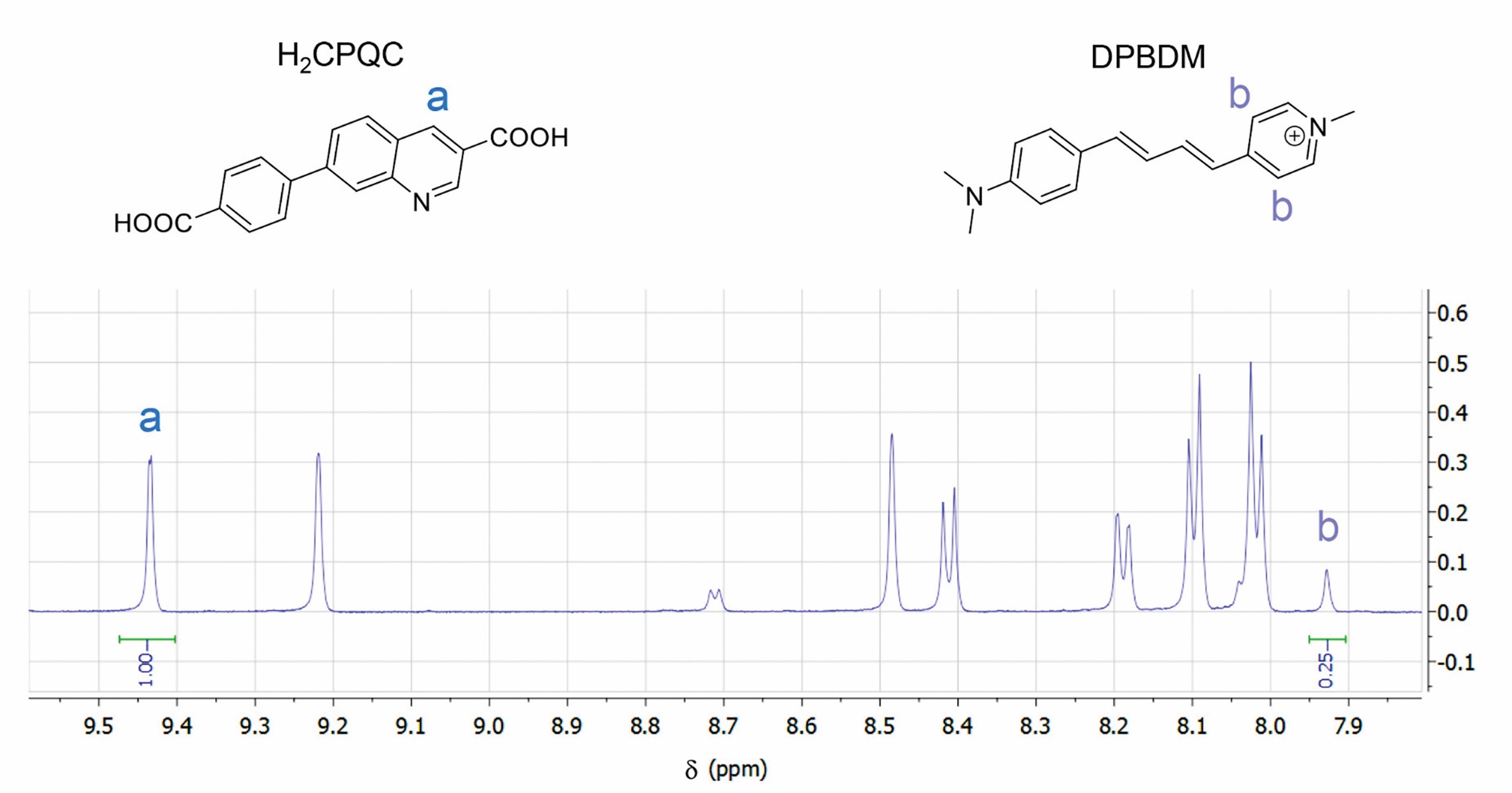


**Figure S4.** The partial ^1^H NMR spectrum of ZJU-68⊃DPBDM crystal segments. We calibrated and obtained peak area values of peaks that belong to linker H_2_CPQC and dye molecule DPBDM, respectively. The ratio (*R*_a_) of their peak area values represents the ratio of their concentrations in the crystal. The dye concentration of the ZJU-68⊃DPBDM crystal segment is calculated to be ~8.49 wt% (~0.53 M).


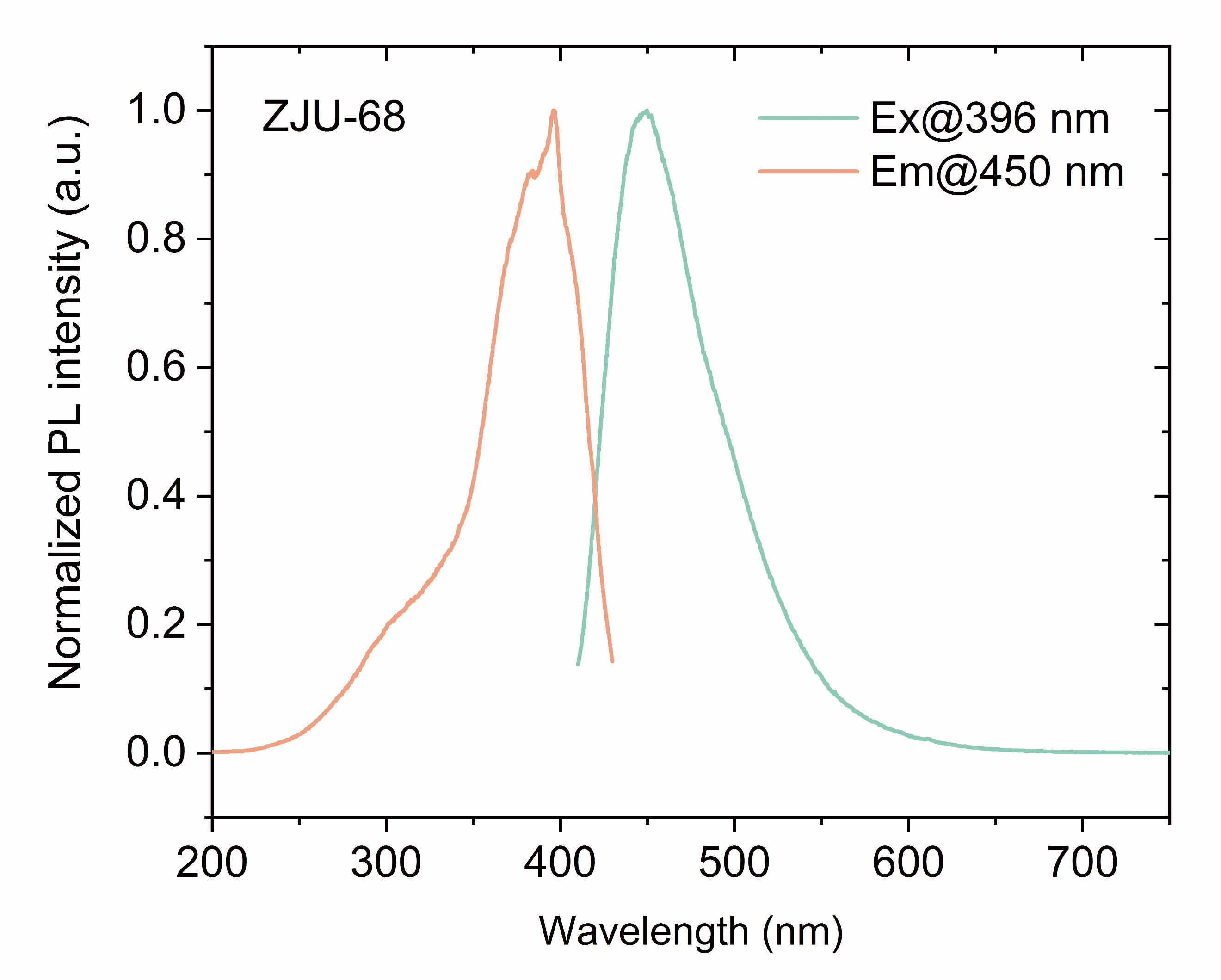


**Figure S5.** The excitation-emission spectra of ZJU-68, showing the maximum excitation position is at 396 nm, and the maximum emission is at 450 nm.


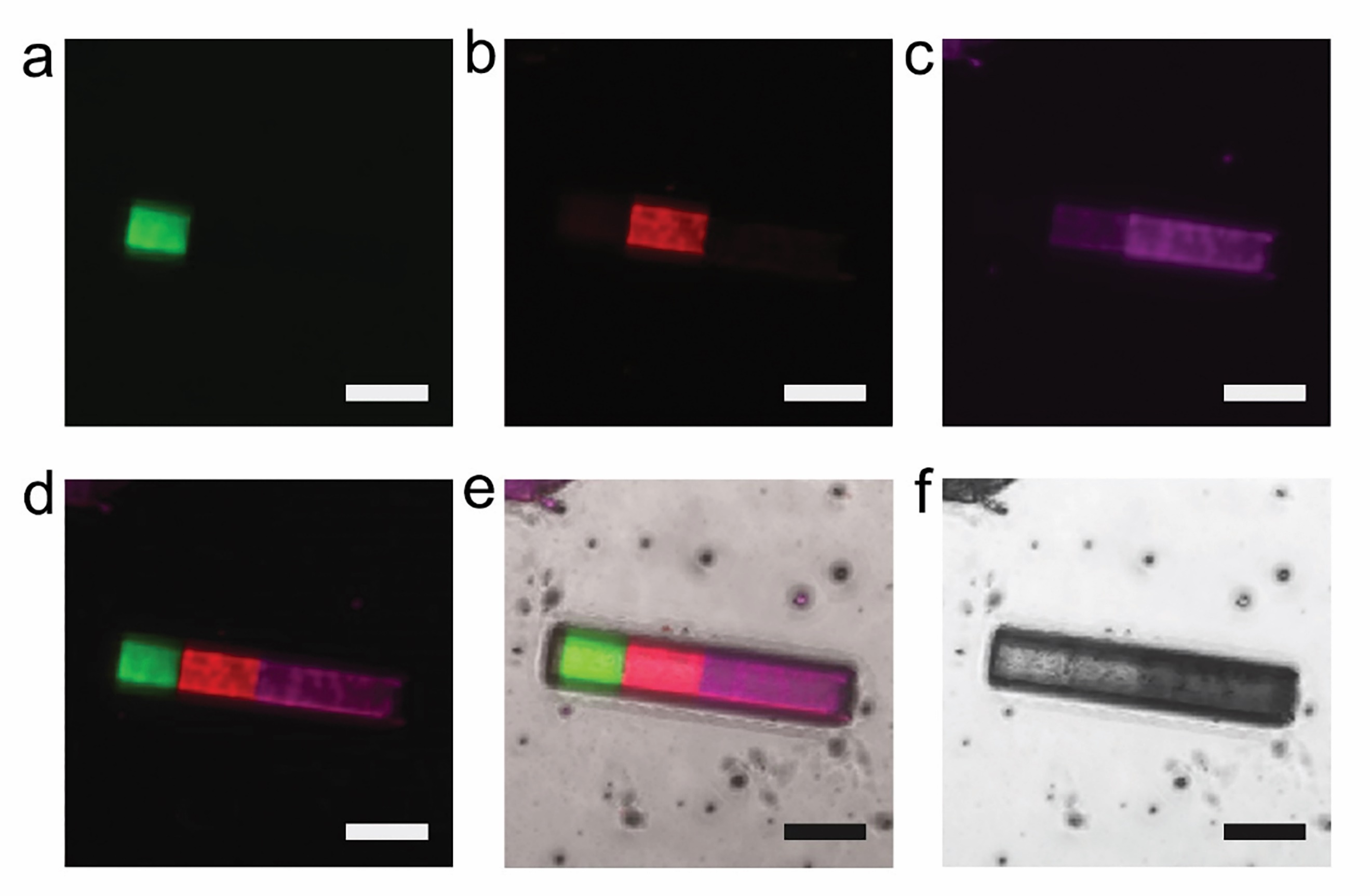


**Figure S6.** The fluorescence micrographs of a single microcrystal ZJU-68⊃DPBDM+DMASM+MMPVP taken by confocal laser scanning microscope. (a) Single channel fluorescence micrograph of ZJU-68⊃DPBDM+DMASM+MMPVP with excitation wavelength of 488 nm and emission filter of 500-550 nm. (b) Single channel fluorescence micrograph of ZJU-68⊃DPBDM+DMASM+MMPVP with excitation wavelength of 559 nm and emission filter of 600-650 nm. (c) Single channel fluorescence micrograph of ZJU-68⊃DPBDM+DMASM+MMPVP with excitation wavelength of 633 nm and emission filter of 650-750 nm. (d) Three channels fluorescence micrograph of ZJU-68⊃DPBDM+DMASM+MMPVP with excitation wavelength combination of 488 nm, 559 nm and 633 nm. (e) Four channels fluorescence micrograph of ZJU-68⊃DPBDM+DMASM+MMPVP with excitation wavelength combination (488+559+633 nm) and transmitted-light detection (TD). (f) The TD channel micrograph of ZJU-68⊃DPBDM+DMASM+MMPVP. Scale bar, 10 μm.


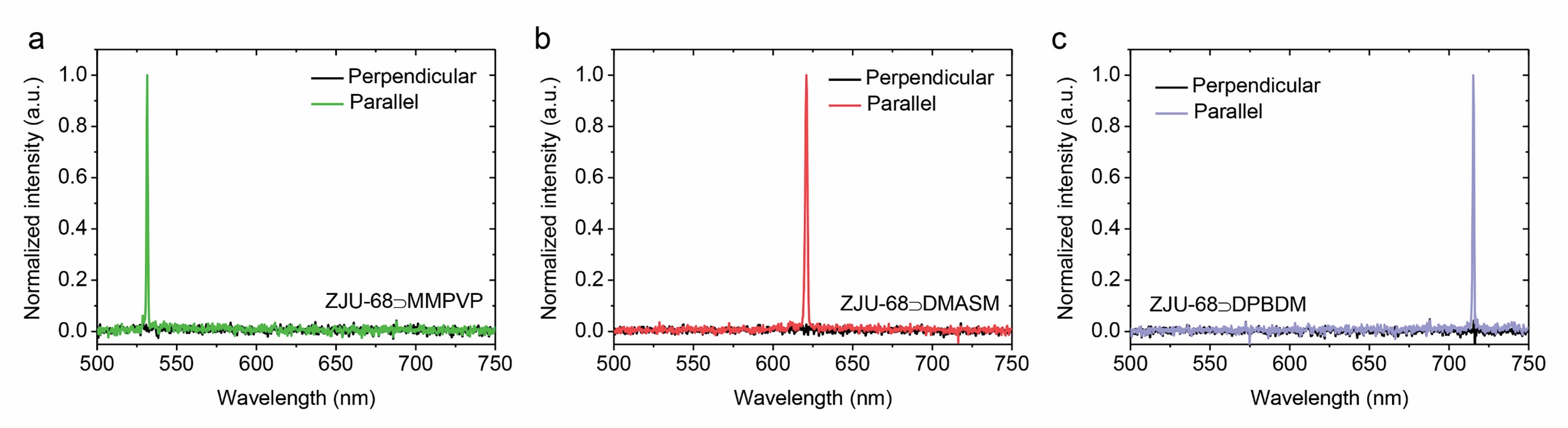


**Figure S7.** The anisotropic lasing performance of the dye-assembled ZJU-68 hybrid microcrystal. (a-c) Intensity-dependent emission spectra from an individual dye-assembled microcrystal ZJU-68⊃MMPVP (a), ZJU-68⊃DMASM (b), and ZJU-68⊃DPBDM (c) respectively with emission-detected polarization at two angles *θ* = 0° (parallel to the crystal channels) and *θ* = 90° (perpendicular to the crystal channels), excited at 480 nm.

**Table S1.** Comparison of lasing thresholds in the reported multi-color lasing monomer materials.

| Materials | Lasing performance | Pump source | | | Thresholds | | |
| --- | --- | --- | --- | --- | --- | --- | --- |
|  |  | Wave-  length | Pulse width | Repetition frequency | Energy density | Power density | Peak power density |
| ZnCdSSe nanosheet^1^ | 530-675 nm three-color | 355 nm | 9 ns | 10 Hz | 11.79 mJ cm^-2^ | 117.9 mW cm^-2^ | 1.31  MW cm^-2^ |
| CdS_x_Se_1-x_CdS nanoribbon^2^ | 517-608 nm dual color | 355 nm | No mention | No mention | - | - | 200  kW cm^-2^ |
| CdSSe nanowire^3^ | 530-637 nm dual color | 355 nm | 9 ns | 10 Hz | 78.3  μJ cm^-2^ | 783  μW cm^-2^ | 8.7  kW cm^-2^ |
| CdSSe nanosheet^4^ | 526-623 nm dual color | 355 nm | 9 ns | 10 Hz | 2.7  mJ cm^-2^ | 27  mW cm^-2^ | 300  kW cm^-2^ |
| CsPbBr_x_I_3-x_ graded nanowire^5^ | 510-556 nm dual color | 400 nm | 150 fs | 1 kHz | 28  μJ cm^-2^ | 28  mW cm^-2^ | 187  MW cm^-2^ |
| RhB-uranin core-shell heterostructure^6^ | 540-610 nm dual color | 400 nm | 200 fs | 1 kHz | 51.5  μJ cm^-2^ | 51.5  mW cm^-2^ | 258  MW cm^-2^ |
| DASPI&LDS 750@rho-ZMOF microcrystal^7^ | 600-740 nm dual color | 532 nm | 150 fs | 1 kHz | 14.0  μJ cm^-2^ | 14.0  mW cm^-2^ | 93  MW cm^-2^ |
| DSM&DABP@bio-MOF-1-2Me microcrystal^8^ | 600-700 nm dual color | 532 nm | ~400 fs | 1 kHz | ~1.80 mJ cm^-2^ | ~1.80  W cm^-2^ | ~4.50 GW cm^-2^ |
| ZJU-68⊃DPBDM+DMASM+MMPVP microcrystal (this work) | 534-720 nm three color | 480 nm | ~400 fs | 1 kHz | ~1.72 mJ cm^-2^ | ~1.72  W cm^-2^ | ~4.30 GW cm^-2^ |

**Table S2.** Comparison of lasing performance in reported multi-color lasing monomer materials.

| Materials | Lasing wavelength coverage | Lasing modes | Lasing line width | Polarization properties |
| --- | --- | --- | --- | --- |
| ZnCdSSe nanosheet^1^ | 145 nm | Multi-modes | 0.4 nm | No mention |
| CdS_x_Se_1-x_CdS nanoribbon^2^ | 91 nm | Single-mode | ~0.7 nm | No mention |
| CdSSe nanowire^3^ | 107 nm | Multi-modes | 0.9 nm | No mention |
| CdSSe nanosheet^4^ | 97 nm | Multi-modes | ~0.2 nm | No mention |
| CsPbBr_x_I_3-x_ graded nanowire^5^ | 46 nm | Multi-modes | 0.3~0.4 nm | No mention |
| RhB-uranin core-shell heterostructure^6^ | 70 nm | Multi-modes | 0.6 nm | No mention |
| DASPI&LDS 750@rho-ZMOF microcrystal^7^ | 140 nm | Multi-modes | No mention | No mention |
| DSM&DABP@bio-MOF-1-2Me microcrystal^8^ | 100 nm | Multi-modes | 0.90~1.07 nm | No mention |
| ZJU-68⊃DPBDM+DMASM+MMPVP microcrystal (this work) | 186 nm | Single-mode | 0.68~0.84 nm | Linear polarization with degree of polarization > 99.9% |

**Supplementary Methods**

**Materials synthesis**

**Synthesis of ZJU-68⊃MMPVP.** A mixture of MMPVP iodide (5.44 μmol, ~1.8 mg), Zn(BF_4_)_2_·xH_2_O (0.068 mmol, ~20 mg), H_2_CPQC (0.034 mmol, 10 mg), DMF (2 mL), MeCN (0.4 mL) and H_2_O (0.01 mL) was sealed in a 15 mL Teflon-lined stainless-steel bomb, subjected to ultrasonic vibration for 5 minutes, which was then heated at 100 °C for 24 h, and cooled to room temperature after reaction. After decanting the mother liquor, the yellow hexagonal crystalline product was rinsed four times with fresh DMF (5 mL × 4) and dried in air.

**Synthesis of ZJU-68⊃DMASM.** A mixture of DMASM iodide (5.44 μmol, ~2.0 mg), Zn(BF_4_)_2_·xH_2_O (0.068 mmol, ~20 mg), H_2_CPQC (0.034 mmol, 10 mg), DMF (2 mL), MeCN (0.4 mL) and H_2_O (0.01 mL) was sealed in a 15 mL Teflon-lined stainless-steel bomb, subjected to ultrasonic vibration for 5 minutes, which was then heated at 100 °C for 24 h, and cooled to room temperature after reaction. After decanting the mother liquor, the red hexagonal crystalline product was rinsed four times with fresh DMF (5 mL × 4) and dried in air.

**Synthesis of ZJU-68⊃DPBDM.** A mixture of DPBDM iodide (5.44 μmol, ~2.1 mg), Zn(BF_4_)_2_·xH_2_O (0.068 mmol, ~20 mg), H_2_CPQC (0.034 mmol, 10 mg), DMF (2 mL), MeCN (0.4 mL) and H_2_O (0.01 mL) was sealed in a 15 mL Teflon-lined stainless-steel bomb, subjected to ultrasonic vibration for 5 minutes, which was then heated at 100 °C for 24 h, and cooled to room temperature after reaction. After decanting the mother liquor, the purple hexagonal crystalline product was rinsed four times with fresh DMF (5 mL × 4) and dried in air.

**Synthesis of ZJU-68⊃DMASM+MMPVP.** A mixture of DMASM iodide (5.44 μmol, ~2.0 mg), Zn(BF_4_)_2_·xH_2_O (0.068 mmol, ~20 mg), H_2_CPQC (0.034 mmol, 10 mg), DMF (2 mL), MeCN (0.4 mL) and H_2_O (0.01 mL) was sealed in a 15 mL Teflon-lined stainless-steel bomb, subjected to ultrasonic vibration for 5 minutes, which was then heated at 100 °C for 24 h. After cooling to room temperature, the reacted solution was carefully removed, the red crystals were washed three times with fresh DMF, and the DMF wash was thoroughly removed. A mixture of MMPVP iodide (5.44 μmol, ~1.8 mg), Zn(BF_4_)_2_·xH_2_O (0.068 mmol, ~20 mg), H_2_CPQC (0.034 mmol, 10 mg), DMF (2 mL), MeCN (0.4 mL) and H_2_O (0.01 mL) was sealed in the above Teflon-lined stainless-steel bomb containing the washed red crystal product, which was then heated at 100 °C for 6 h. After cooling to room temperature and decanting the mother liquor, the red-yellow segmented hexagonal crystalline product was rinsed four times with fresh DMF (5 mL × 4) and dried in air.

**Synthesis of ZJU-68⊃DPBDM+DMASM.** A mixture of DPBDM iodide (5.44 μmol, ~2.1 mg), Zn(BF_4_)_2_·xH_2_O (0.068 mmol, ~20 mg), H_2_CPQC (0.034 mmol, 10 mg), DMF (2 mL), MeCN (0.4 mL) and H_2_O (0.01 mL) was sealed in a 15 mL Teflon-lined stainless-steel bomb, subjected to ultrasonic vibration for 5 minutes, which was then heated at 100 °C for 24 h. After cooling to room temperature, the reacted solution was carefully removed, the purple crystals were washed three times with fresh DMF, and the DMF wash was thoroughly removed. A mixture of DMASM iodide (5.44 μmol, ~2.0 mg), Zn(BF_4_)_2_·xH_2_O (0.068 mmol, ~20 mg), H_2_CPQC (0.034 mmol, 10 mg), DMF (2 mL), MeCN (0.4 mL) and H_2_O (0.01 mL) was sealed in the above Teflon-lined stainless-steel bomb containing the washed purple crystal product, which was then heated at 100 °C for 6 h. After cooling to room temperature and decanting the mother liquor, the purple-red segmented hexagonal crystalline product was rinsed four times with fresh DMF (5 mL × 4) and dried in air.

**Synthesis of ZJU-68⊃DPBDM+MMPVP.** A mixture of DPBDM iodide (5.44 μmol, ~2.1 mg), Zn(BF_4_)_2_·xH_2_O (0.068 mmol, ~20 mg), H_2_CPQC (0.034 mmol, 10 mg), DMF (2 mL), MeCN (0.4 mL) and H_2_O (0.01 mL) was sealed in a 15 mL Teflon-lined stainless-steel bomb, subjected to ultrasonic vibration for 5 minutes, which was then heated at 100 °C for 24 h. After cooling to room temperature, the reacted solution was carefully removed, the purple crystals were washed three times with fresh DMF, and the DMF wash was thoroughly removed. A mixture of MMPVP iodide (5.44 μmol, ~1.8 mg), Zn(BF_4_)_2_·xH_2_O (0.068 mmol, ~20 mg), H_2_CPQC (0.034 mmol, 10 mg), DMF (2 mL), MeCN (0.4 mL) and H_2_O (0.01 mL) was sealed in the above Teflon-lined stainless-steel bomb containing the washed purple crystal product, which was then heated at 100 °C for 6 h. After cooling to room temperature and decanting the mother liquor, the purple-yellow segmented hexagonal crystalline product was rinsed four times with fresh DMF (5 mL × 4) and dried in air.

**Synthesis of ZJU-68⊃DPBDM+DMASM+MMPVP.** A mixture of DPBDM iodide (5.44 μmol, ~2.1 mg), Zn(BF_4_)_2_·xH_2_O (0.068 mmol, ~20 mg), H_2_CPQC (0.034 mmol, 10 mg), DMF (2 mL), MeCN (0.4 mL) and H_2_O (0.01 mL) was sealed in a 15 mL Teflon-lined stainless-steel bomb, subjected to ultrasonic vibration for 5 minutes, which was then heated at 100 °C for 24 h. After cooling to room temperature, the reacted solution was carefully removed, the purple crystals were washed three times with fresh DMF, and the DMF wash was thoroughly removed. A mixture of DMASM iodide (5.44 μmol, ~2.0 mg), Zn(BF_4_)_2_·xH_2_O (0.068 mmol, ~20 mg), H_2_CPQC (0.034 mmol, 10 mg), DMF (2 mL), MeCN (0.4 mL) and H_2_O (0.01 mL) was sealed in the above Teflon-lined stainless-steel bomb containing the washed purple crystal product, which was then heated at 100 °C for 6 h. After cooling to room temperature, the reacted solution was carefully removed, the purple-red crystals were washed three times with fresh DMF, and the DMF wash was thoroughly removed. A mixture of MMPVP iodide (5.44 μmol, ~1.8 mg), Zn(BF_4_)_2_·xH_2_O (0.068 mmol, ~20 mg), H_2_CPQC (0.034 mmol, 10 mg), DMF (2 mL), MeCN (0.4 mL) and H_2_O (0.01 mL) was sealed in the above Teflon-lined stainless-steel bomb containing the washed purple-red crystal product, which was then heated at 100 °C for 6 h. After cooling to room temperature and decanting the mother liquor, the purple-red-yellow segmented hexagonal crystalline product was rinsed four times with fresh DMF (5 mL × 4) and dried in air.

**Measurements**

For material characterization, powder X-ray diffraction (PXRD) patterns were collected in the 2*θ* = 5~40° range on an X’Pert PRO diffractometer with Cu Kα radiation (*λ* = 1.542 Å) at room temperature.

Micrographs and microscopic spectra were taken on an Olympus IX71 inverted fluorescence microscope. Confocal laser scanning images were taken on an Olympus FV1000 laser scanning confocal microscope equipped with an Olympus IX81 inverted microscope. The excitation-emission spectra of ZJU-68 in Figure S5 were taken by the Edinburgh Instrument F900 fluorescence spectrometer.

For lasing experiments, an optical parametric amplifier (Spirit-OPA + Spirit-OPA-UV3, Newport Corporation) was pumped by a fully automated ultrafast laser system (Spirit One 1040-8, 8 W at 1040 nm, Newport Corporation), which was used for generating the excitation pulse (1 kHz, 480 nm, pulse width < 400 fs). The incident laser was coupled to a microscope (IX71, Olympus) for focusing on crystals through an objective lens (Olympus LUCPlanFL N 40×, numerical aperture = 0.60). The emission light was then focused and collected by the fibre optic spectrometer (PG2000-Pro, Ideaoptics Instruments).

**Determination of dye concentrations.**

Concentrations of well-dried dye-included ZJU-68 hybrid crystals were determined by ^1^H NMR. As shown in Figure S2 to Figure S4, we calibrated and obtained peak area values of peaks that belong to dyes (MMPVP or DMASM or DPBDM) and H_2_CPQC, respectively. The ratio (*R*_a_) of their peak area values represents the ratio of their concentrations in the crystal. The dye concentration of dye-included ZJU-68 hybrid crystals is calculated from *c* = 3*R*_a_/*N*_A_*V*, where *V* = 2403.91 Å^3^ and *N*_A_ = 6.02×10^23^ mol^-1^ is Avogadro’s constant.

**References**

1 Fan, F. *et al*. A monolithic white laser. *Nature Nanotechnology* **10**, 796-803 (2015).

2 Xu, J. Y. *et al*. Room-temperature dual-wavelength lasing from single-nanoribbon lateral heterostructures. *Journal of the American Chemical Society* **134**, 12394-12397 (2012).

3 Liu, Z. C. *et al*. Dynamical color-controllable lasing with extremely wide tuning range from red to green in a single alloy nanowire using nanoscale manipulation. *Nano Letters* **13**, 4945-4950 (2013).

4 Fan, F. *et al*. Simultaneous two-color lasing in a single CdSSe heterostructure nanosheet. *Semiconductor Science and Technology* **28**, 065005 (2013).

5 Huang, L. *et al*. Composition-graded cesium lead halide perovskite nanowires with tunable dual-color lasing performance. *Advanced Materials* **30**, 1800596 (2018).

6 Zhou, Z. H. *et al*. Organic printed core-shell heterostructure arrays: a universal approach to all-color laser display panels. *Angewandte Chemie International Edition* doi: 10.1002/anie.202002580 (2020).

7 Zhang, Y. *et al*. Dual-wavelength lasing from organic dye encapsulated metal-organic framework microcrystals. *Chemical Communications* **55**, 3445-3448 (2019).

8 Li, H. J. *et al*. Dual-band simultaneous lasing in MOFs single crystals with Fabry-Perot microcavities. *Science China Chemistry* **62**, 987-993 (2019).
